# Transcriptome analysis reveals the key long non-coding RNAs and genes related to cashmere shedding in goats

**DOI:** 10.5713/ab.25.0499

**Published:** 2025-10-22

**Authors:** Yixing Fan, Tiantian Gong, Siyu Feng, Huiling Zhang, Ran Wang, Mingzhao Hu, Qi Zhang, Taiyu Hui, Jincheng Shen, Ruqing Xu, Yubo Zhu, Man Bai, Zeying Wang, Wenlin Bai

**Affiliations:** 1 College of Animal Science & Veterinary Medicine, Shenyang Agricultural University, Shenyang, China; 2 Engineering Research Center for Animal Molecular Genetics and Breeding of Liaoning Province, Shenyang, China; 3 Laboratory Animal Center, General Hospital of Northern Theater Command, Shenyang, China; 4 Shenyang Jinghua Hospital, Shenyang, China

**Keywords:** Cashmere Goat, Cashmere Shedding, Hair Follicle Regeneration, *LOC108637151*, Long Non-coding RNA, *SEPP1*

## Abstract

**Objective:**

The cashmere goat is renowned for the exceptional quality of cashmere fibers. The shedding of cashmere is closely related to the production processes in cashmere goats, but its molecular regulatory mechanism is not fully understood.

**Methods:**

In this study, we collected skin tissues from both already-shed cashmere goats (AS) and non-shed cashmere goats (NS). Morphological differences were observed, and the relative expression levels of indicator genes distinguishing anagen from telogen phases of hair follicles were assessed. Whole transcriptome sequencing was employed to investigate key regulatory factors including long non-coding RNAs (lncRNAs) and different expression genes (DEGs) and followed by preliminary validation, interaction network construction and functional verification.

**Results:**

Comparative histological analysis found that the density, depth, width, hair bulb width and activity of secondary hair follicles (SHFs) in AS individuals were significantly lower than those in NS individuals. Expression detection results of indicator genes distinguishing anagen from telogen indicated that the SHFs of NS individuals were predominantly in telogen phase, whereas those of AS individuals showed a greater tendency towards anagen phase. Transcriptome sequencing analysis identified 450 DEGs with 338 upregulated and 112 downregulated. as Additionally, 352 lncRNAs with different expression were detected, including 168 upregulated and 184 downregulated. Regulatory networks involving lncRNAs and their co-expressed DEGs were established. The lncRNA *LOC108637151* and its co-expression gene, Selenoprotein P (*SEPP1*), were identified as key regulatory factors of cashmere shedding in goats, both exhibiting elevated expression levels in AS individuals. The overexpression of *LOC108637151* in dermal papilla cells (DPCs) resulted in the increased expression of its target gene *SEPP1* and promoted the proliferation of DPCs in cashmere goats.

**Conclusion:**

This study identified key lncRNAs and genes related to cashmere shedding in goats, as well as their regulatory relationships. The results provided a basis for revealing the potential molecular mechanisms underlying cashmere shedding in goats.

## INTRODUCTION

Cashmere goat is a vital economic animal in China, and is renowned for its superior characteristics of cashmere fibers. Liaoning cashmere goat, an exemplary local breed in China, is considered a national treasure. Cashmere is produced from the secondary hair follicles (SHFs) in skin tissue of cashmere goats. The SHFs are complex skin accessory organs that develop from embryonic epithelial cells and regulate cashmere fiber growth [1]. The growth and development of hair follicles occur in periodic manner, with the initiation of hair follicle formation being asynchronous [2]. Cashmere goats exhibit periodic hair follicle growth. For instance, the growth cycle of SHFs of Liaoning cashmere goats can be delineated into five stages: early anagen (May to July), anagen (August to December), early catagen (January), catagen (February), and telogen (March to April) [3].

Hair follicles undergo active growth during anagen phase, whereas they remain in a resting state during telogen phase. Catagen phase serves as a relatively short transitional period between anagen and telogen [4,5]. Due to the cyclical growth patterns of hair follicles, cashmere goats undergo significant seasonal shedding of cashmere fiber with the timing of shedding and regeneration on the body surface varying. Cashmere shedding commences from late telogen to early anagen, typically occurring from April to May each year, and new hair emerges on the body surface between July and August. Through extensive observation, researchers have identified patterns in the cashmere shedding times of cashmere goats under uniform raising conditions, within same breed: older goats tend to shed earlier than younger ones; female individuals shed earlier than male individuals; goats with better nutritional status shed earlier. Additionally, the timing of cashmere shedding varies across different body parts of the same goat with forequarters shedding before the hindquarters [6].

Long non-coding RNA (lncRNA) is a class of non-coding RNA (ncRNA) that exceeds 200 nucleotides in length, and it shares characteristics with mRNA, except for its lack of coding potential [7,8]. Recent studies have demonstrated that lncRNA could regulate gene expression at both the transcriptional and post-transcriptional levels [9,10], thereby influencing various traits in farm animals. To date, however, little information is available on the regulator effects of lncRNAs on cashmere shedding in goats.

In this study, we investigated the variations in cashmere shedding time among cashmere goats with a comprehensive analysis of underlying mechanism of the differences through transcriptome sequencing analysis followed by functional validation in dermal papilla cells (DPCs) *in vitro*. This study aims to provide a theoretical foundation for further revealing the molecular mechanisms understanding the cashmere shedding in goats.

## MATERIALS AND METHODS

### Sample collection

**Figure 1:**
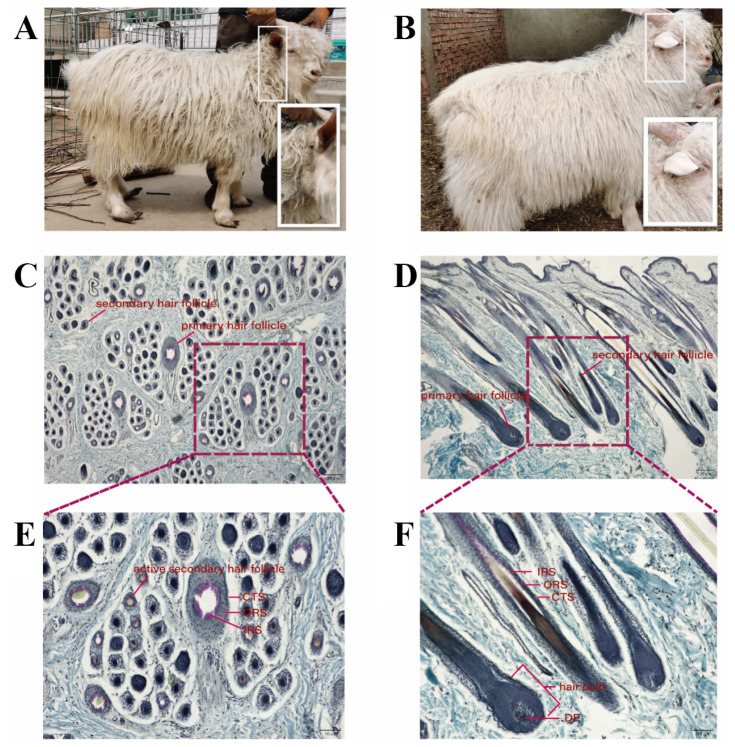
Already shed (AS) cashmere goat and non-shed (NS) cashmere goat in early April, as well as the photomicrographs of skin tissues. (A) AS male cashmere goat where the cashmere at its neck had fallen off and adhered to the hair. (B) NS male cashmere goat where cashmere at neck was still firmly ingrained in the skin. (C–F) Photomicrographs of skin tissues of cashmere goats (c and d Sacpic method, ×40; e and f Sacpic method, ×100). The abbreviations in the figure stand for connective tissue sheath (CTS), outer root sheath (ORS), inner root sheath (IRS), hair shaft (HS), dermal papilla (DP) and hair bulb.

At the beginning of April, the cashmere goat herd gradually began to shed cashmere. In this experiment, the group goats with cashmere on their neck that has fallen off and adhered to the hair would be considered as already-shed (AS) cashmere goats (Figure 1A), while the other group goats, whose neck cashmere is still firmly ingrained in the skin, would be considered as non-shed (NS) cashmere goats (Figure 1B).

Three AS and three NS male cashmere goats, maintained under the same feeding and management conditions, were selected in this study. Skin tissues were collected from the lateral body region of these goats. The collected skin tissue was divided into two equal parts. One part was immediately fixed in 4% paraformaldehyde for 24 h, then dehydrated using a gradient ethanol series to 75% ethanol, and subsequently stored at −20°C for tissue sectioning. The other part was promptly placed in a liquid nitrogen and stored at −80°C for total RNA extraction with transcriptome sequencing and subsequent validation.

### Preparation of skin tissue paraffin section

The skin tissues of AS and NS cashmere goats were fixed in 4% paraformaldehyde for 24 h to prepare tissue sections. After washing with phosphate-buffered saline (PBS), the tissues were dehydrated using a gradient ethanol series, cleared with xylene, and then were embedded in paraffin. For the transverse and longitudinal sections, each paraffin-embedded sample was serially sectioned at a thickness of 6 μm using a rotary microtome (HM355S, Thermo Fisher Scientific) and subsequently stained using a modified Sacpic method [11].

### Determination of parameters related to hair follicle groups

The well-stained transverse and longitudinal paraffin sections from two groups were examined and photographed using a microscope (OLYMPUS CX43RF) and camera (Mshot MSX2), respectively. For each sample, 30 microscopic fields from the sections were observed, and statistical analyses were conducted on both transverse and longitudinal sections. This included measurements of the depth of primary hair follicles (PHFs) and SHFs in micrometers (μm), as well as the width of SHFs. The number of PHFs, total SHFs, and active SHFs in 30 different transverse section fields of view were counted. Subsequently, the density of PHFs (N/μm^2^), density of SHFs (N/μm^2^), activity of SHFs (%), and the ratio of active SHFs to PHFs (active S/P) were calculated. The depth of PHFs and SHFs, along with the width of the hair bulb of the SHFs, were observed and analyzed across 30 different longitudinal section fields.

### Whole transcriptome analysis

Skin tissues from AS and NS cashmere goats, stored at −80°C, were utilized for whole transcriptome sequencing analysis. RNA isolation, library construction, RNA sequencing, and bioinformatics analysis were performed by Novogene. Sequence data that support the findings of this study had been deposited in the GenBank Sequence Read Archive (SRA) database under accession number PRJNA1227581. Transcriptome data were compared to the goat reference genome (*ARS 2.0*) for comparative analysis. Ten different expression genes (DEGs; *C7, DCLK1, THFAIP6, DUSP1, ATF3, FAT4, FHL1, PLN, SERP4* and *GREM1*) were randomly selected to verify their relative expression levels of two groups.

Reads were spliced into transcripts and quantified based on the results of genome alignment using Stringtie [12,13]. The naming of lncRNA was performed through referring to HGNC (The HUGO Gene Nomenclature Committee) for guidance, and novel lncRNA candidates were finally screened and named according to their position relationship with coding genes. After the above alignment, splicing and screening, the expression levels of the mRNA, novel lncRNA, novel mRNA and unclassified transcripts were quantified, and the results were expressed as FPKM values with the difference significance analysis. The Goseq [14] and KOBAS (2.0) [15] were used to perform the Gene Ontology (GO) enrichment and pathway enrichment analysis of DEGs. Cytoscape 3.10.1 [16] was used to construct the interaction network of DEGs and their co-expression different lncRNAs.

### Reverse transcription quantitative polymerase chain reaction and Western-blotting analysis

Reverse transcription quantitative polymerase chain reaction (RT-qPCR) was used to detect the different expression level of indicator genes and DEGs in the analyzed samples. All the primers (Supplement 1) used in RT-qPCR was designed and synthesized by Sangon Biotech. The total RNA was isolated from skin tissue using the RNAiso Plus (Takara Bio). RT-qPCR reactions were performed by the PrimeScript RT Master Mix along with TB Green Premix Ex Taq (Tli RNaseH Plus) assay (Takara Bio). Relative expression levels of mRNA and lncRNA were calculated by the method of 2*^−ΔΔct^*.

The proteins extracted from skin tissue were quantified using the bicinchoninic acid method (BCA protein assay) and adjusted to the same concentration as the candidate proteins. Proteins were loaded onto the SDS-PAGE gel, and analyzed by Western-blotting technique using antibodies (BMP4: A11315, ABclonal) against the tested protein. Results were visualized using a high-sensitivity chemiluminescence detection kit (Epizyme Biotech). The X-ray film was exposed for developing and photographing.

### Cell culture and transfection

DPCs were cultured in Dulbecco’s Modified Eagle Medium/Nutrient Mixture F-12 (DMEM/F12; HyClone) supplemented with 10% fetal bovine serum (FBS; ExCell Bio) and 1% penicillin-streptomycin (Gibco) at 37°C in a 5% CO_2_ incubator. The overexpression plasmid pcDNA3.1-*LOC108637151* and the negative control plasmid pcDNA3.1-NC were obtained from Shanghai Sangon Biotech. Prior to plasmid transfection, DPCs exhibiting optimal growth were seeded in 6-well plates and maintained in DMEM supplemented with 10% FBS (HyClone) under the same incubation conditions. Upon reaching approximately 70% confluence, the pcDNA3.1-*LOC108637151* overexpression vector was transfected into the DPCs with a concentration of 1 μg for a duration of 8 h. RT-qPCR was employed to assess the expression level of *LOC108637151*. The expression levels of *SEPP1* were subsequently evaluated for confirming transfection efficiency. The expression of proliferation markers *Ki67* and *PCNA* was analyzed for the assessment of cell proliferation. Transfected DPCs were seeded into 96-well plates at a density of 1.5×10^5^ cells per well and cultured for 24 h, after which 10 μL of Cell Counting Kit-8 (CCK-8; Vazyme) solution was added to each well. Each experiment was replicated three times.

### Statistical analysis

The obtained data was analyzed by SPSS 27.0 and GraphPad Prism 9.0 procedures. Results were expressed as mean value± standard deviation. Data on the cashmere performances and parameters related to hair follicle was analyzed by Duncan test. Difference at p<0.05 was considered to be significant, while p<0.01 was considered to be extremely significant. The same shoulder letters in the same row of data, represent no significant differences, and different shoulder letters represent significant differences.

## RESULTS

### Morphology structure observation of skin tissue in already-shed and non-shed cashmere goats

Photomicrographs of skin tissue from AS and NS cashmere goats were presented in Figures 1C–1F. The fundamental structure of the hair follicle was discernible from the longitudinal section. The outermost layer, the connective tissue sheath (CTS), encased the hair follicle. The outer root sheath (ORS) and inner root sheath (IRS) were within the sheath with the hair shaft (HS) located in the center. The hair bulb, a spherical bulge at the base, contained the dermal papilla (DP) at its center. The diameter of SHFs was narrower, and the HS lacked a medulla.

**Figure 2:**
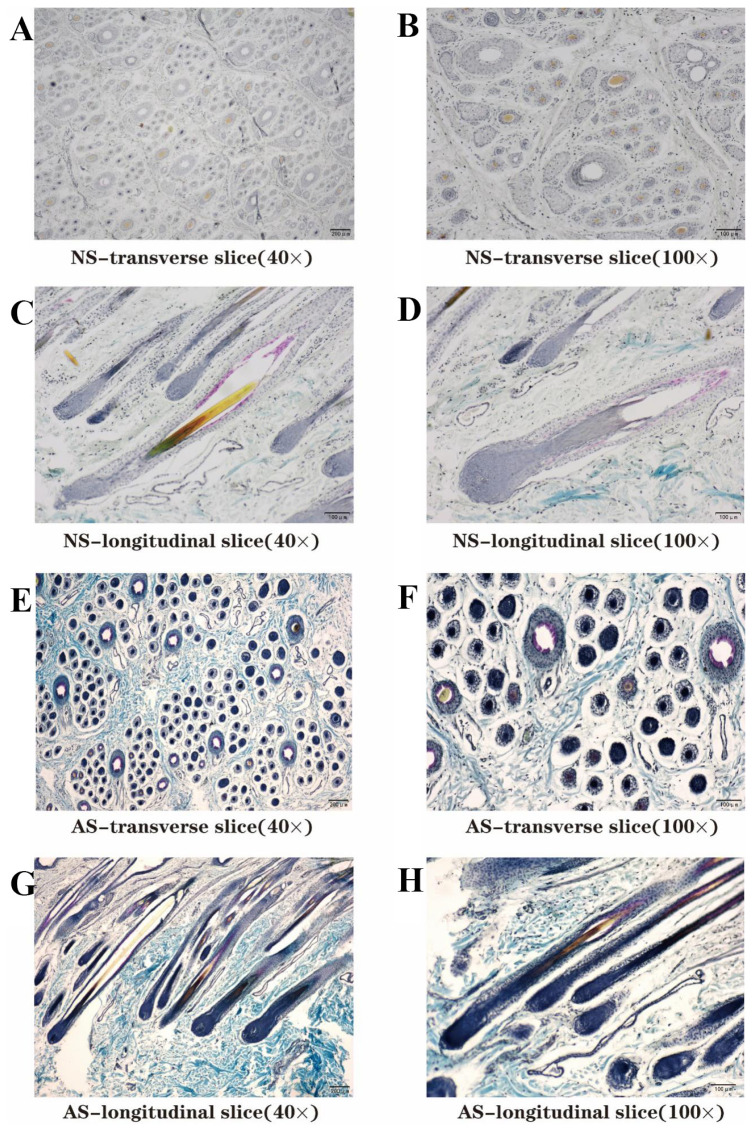
Transverse and longitudinal skin sections of NS and AS cashmere goats. (A–D) NS cashmere goat skin sections. (E–H) AS cashmere goat skin sections (Sacpic method). NS, non-shed; AS, already shed.

Basic morphological structure of skin tissue and hair follicle were evident in the comparative morphological analysis of sections from both groups (Figure 2). However, specific parameters were different between the two groups, such as SHFs density, depth, width, active S/P ratio, active SHF number, and active SHFs density (Figure 2). Upon measurement and statistical analysis of transverse and longitudinal sections, the SHFs density in the AS skin tissue section was found to be 7.10 mm^2^, significantly lower than the 15.89 mm^2^ observed in the NS group. The depth (1,274 μm), the width (58.17 μm), and the active S/P ratio of AS group SHFs was significantly lower than those of NS group (Table 1). The analysis revealed that the depth of SHFs and the width of hair bulbs in AS group were significantly reduced compared to NS group. This suggests that the majority of hair follicles in NS cashmere goats remain in the telogen phase, whereas those in AS cashmere goats are transitioning to the anagen phase.

### Different expression level of indicator genes between anagen and telogen

**Figure 3:**
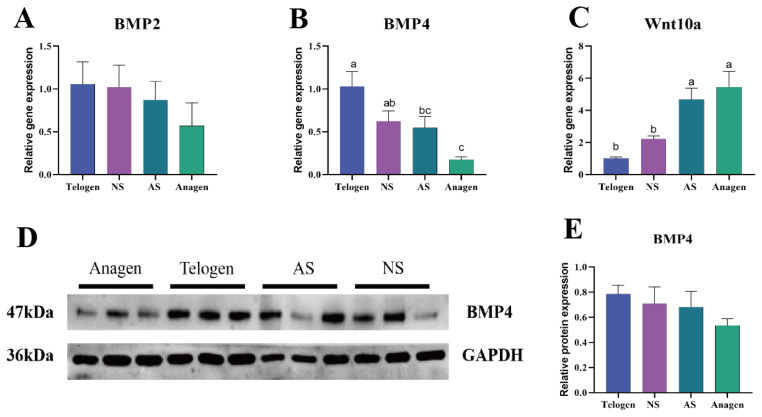
The expression of indicator genes in telogen, NS, AS and anagen tissues. (A–C) Expression of indicator genes include *BMP2*, *BMP4* and *Wnt10a*. (D, E) Expression of BMP4 protein. ^a–c^ Letters on the bars showed the difference among four groups, and different letter was significant, while same letter was non-significant. NS, non-shed; AS, already-shed.

Based on previous studies [17–21], the expression of indicator genes distinguishing anagen from telogen phases (*BMP4*, *BMP2*, and *Wnt10a*) was assessed in telogen, NS, AS, and anagen skin tissues by RT-qPCR technique. Additionally, BMP4 protein expression was evaluated through Western-blotting analysis (Figure 3). Our findings indicated that *BMP2* and *BMP4* expression levels were down-regulated, while *Wnt10a* was up-regulated during tested phases. The relative expression pattern of BMP4 protein was consistent with the RT-qPCR results, aligning with previous studies. Based on the different expression levels of analyzed genes, it was inferred that the SHFs of NS cashmere goats were predominantly in the telogen phase, whereas those of AS cashmere goats were more inclined towards the anagen phase, most possibly initiating a new growth cycle.

### Whole transcriptome analysis

The entire transcriptome dataset was aligned to the goat reference genome (*ARS 1.2*) for comparative analysis. The findings indicated that the unique mapping rates exceeded 86%, suggesting a robust comparison suitable for subsequent analyses (Table 2). Concurrently, a total of 4,832 novel lncRNAs were identified and categorized into three types: antisense (999), lincRNA (2,084), and sense overlapping (1,749) (Supplement 2). A comparative analysis of transcript length, exon count, and open reading frame (ORF) length was conducted between lncRNAs and mRNAs to elucidate their differences and to assess whether the predicted novel lncRNAs align with established characteristics. The results indicated that both annotated and novel lncRNA exhibited a higher density distribution in terms of shorter transcript lengths, fewer exons, and shorter ORFs (Supplement 2).

**Figure 4:**
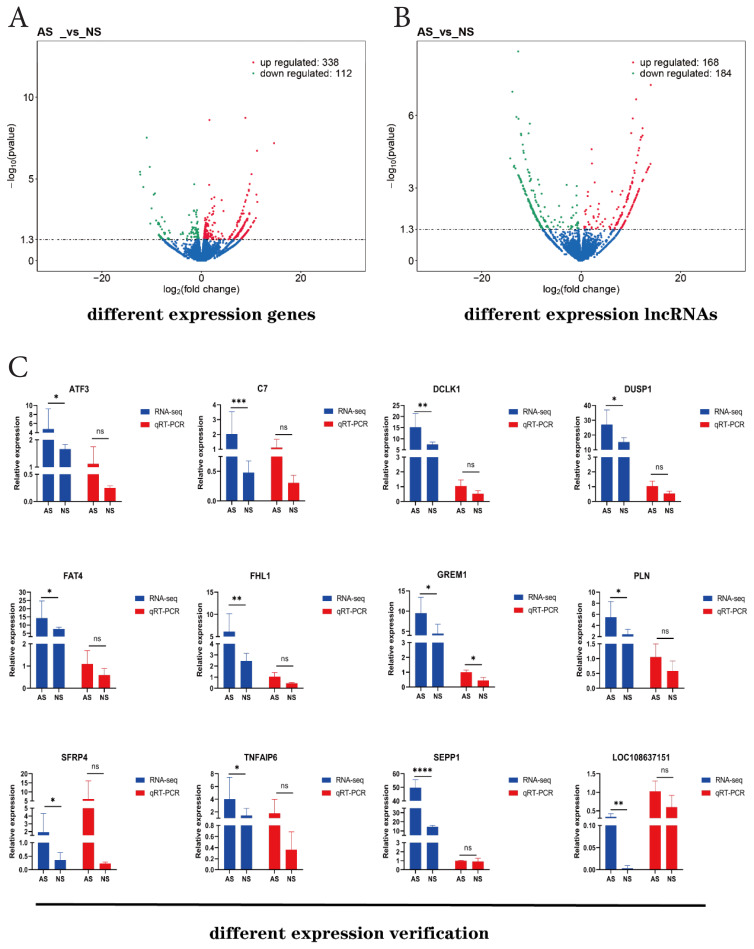
Expression analysis of related genes. (A) Different expression analysis of volcano map of DEGs. (B) Volcano map of lncRNAs. (C) 11 DEGs and lncRNA LOC108637151 for relative expression detection. * p<0.05; ** p<0.01; *** p<0.001; **** p<0.0001; ns, not significant. AS, already-shed; NS, non-shed; qRT-PCR, quantitative reverse transcription polymerase chain reaction; DEGs, different expression genes; lncRNA, long non-coding RNA.

The expression levels of all known transcripts, novel lncRNAs, novel mRNAs, and unclassified transcripts were quantified after comparison, splicing, and screening, and were expressed in fragments per kilobase of transcript per million mapped reads (FPKM). Subsequently, the expression values for each sample were calculated, and differential expression analysis was performed between two groups of goats: AS and NS. A total of 450 DEGs were identified, with 338 exhibiting up-regulation and 112 showing down-regulation. Additionally, 352 different expression lncRNAs were identified, consisting of 168 up-regulated and 184 down-regulated. To verify the accuracy of the sequencing results, we randomly selected 10 DEGs (*C7, DCLK1, THFAIP6, DUSP1, ATF3, FAT4, FHL1, PLN, SERP4* and *GREM1*) for relative expression analysis using RT-qPCR and compared the obtained results with the transcriptomic sequencing data. The results demonstrated that the expression trends of these 10 DEGs were consistent with the sequencing data, confirming the reliability of the sequence results (Figure 4).

**Figure 5:**
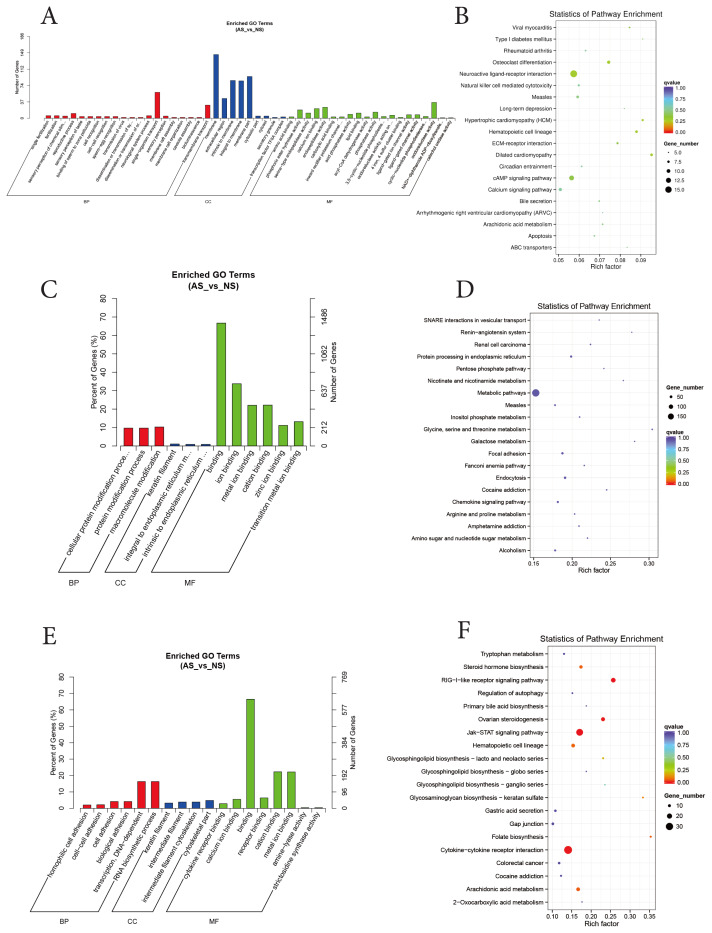
GO and KEGG enrichment analysis. (A, B) GO and KEGG analysis of DEGs. (C, D) GO and KEGG analysis of target genes of co-expression of different expression lncRNAs. (E, F) Target genes of co-location of different expression lncRNAs. GO, Gene Ontology; AS, already-shed; NS, non-shed; KEGG, Kyoto Encyclopedia of Genes and Genomes; DEGs, different expression genes; lncRNA, long non-coding RNA.

Enrichment analysis of DEGs was performed with GO functional and Kyoto Encyclopedia of Genes and Genomes (KEGG) pathway. GO enrichment analysis showed the DEGs were enriched in biological processes (such as single-organism transport and transmembrane transport); cellular component (such as membrane and membrane part); and molecular function (such as oxidoreductase activity and endopeptidase activity). KEGG enrichment analysis showed the DEGs were enriched in neuroactive ligand-receptor interaction, cAMP signaling pathway, osteoclast differentiation and calcium signaling pathway (Figures 5A, 5B). In further to analyze the potential target genes of lncRNAs, we predicted the location and expression between potential target genes and lncRNAs, and tried to explore the molecular mechanisms between lncRNAs and the target mRNAs through co-location and co-expression analysis. To further explore the function of lncRNAs, the target genes that had both co-expression and co-location with the different expression lncRNAs were also analyzed by GO and KEGG enrichment, respectively (Figures 5C–5F). In GO enrichment, the target genes of co-expression mainly were enriched in macromolecule modification and protein modification process of biological processes, in keratin filament and intrinsic to endoplasmic reticulum membrane of cellular component, and in binding and ion binding of molecular function (Figure 5C). In KEGG enrichment, the analyzed genes were mainly enriched in metabolic pathways and Endocytosis (Figure 5D). In the GO enrichment on the target genes co-located with lncRNA were mainly enriched in transcription DNA-dependent and RNA biosynthetic process of biological processes, in cytoskeletal part and intermediate filament cytoskeleton of cellular component, and in binding and cation binding of molecular function (Figure 5E). While In KEGG enrichment, the analyzed genes were mainly enriched in cytokine-cytokine receptor interaction and Jak-STAT signaling pathway (Figure 5F).

### Screening of key different expression genes, different expression long non-coding RNAs and their target genes

**Figure 6:**
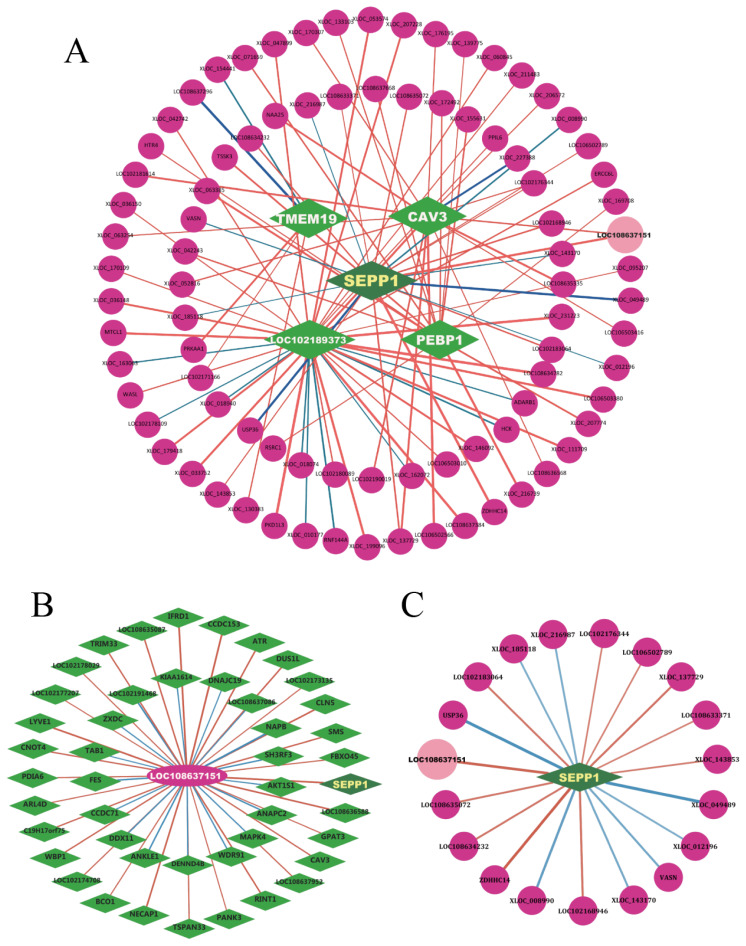
Regulatory network of key lncRNAs and genes potentially related to cashmere shedding in goats. (A) 5 DEGs from top 20 and their 87 co-expression lncRNAs. (B) *LOC108637151* and its co-expression genes. (C) *SEPP1* and its co-expression lncRNAs. DEGs were green diamonds in middle and target lncRNAs were in pink cycle at outside. Red lines between them indicated positive correlation while blue lines indicated negative correlation. Thicker lines represented stronger correlations and thinner lines were the opposite. lncRNA, long non-coding RNA; DEGs, different expression genes.

Based on the differential expression analysis along with enrichment analysis, we constructed a regulatory network including 5 DEGs from the top 20 and their 87 co-expression lncRNAs (Figure 6). Among the top 20 DEGs and lncRNAs with significant differences, we identified the protein-coding gene *SEPP1* (*Selenoprotein P*), and its co-expressed lncRNA-*LOC108637151*. Both *SEPP1* and lncRNA-*LOC108637151* exhibited high expression levels in the AS group according to both sequencing data and RT-qPCR analysis (Figure 4C). We speculate that the interactions between *SEPP1* and lncRNA-*LOC108637151* may affect the variation in cashmere shedding in goats.

### The effect of *LOC108637151* on the proliferation of dermal papilla cells

**Figure 7:**
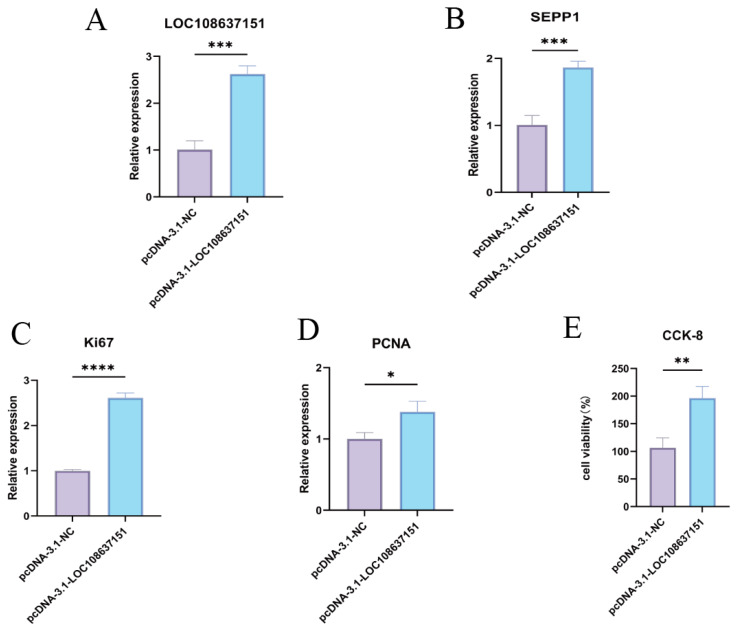
The effect of *LOC108637151* on the proliferation of DPCs. (A) The overexpression of *LOC108637151* in DPCs. (B) Expression of SEPP1 in DPCs. (C, D) Expression of the cell proliferation indicator genes (C) Ki67 and (D) PCNA in DPCs. (E) CCK-8 detection of cell proliferation in DPCs. * p<0.05; ** p<0.01; *** p<0.001; **** p<0.0001. DPC, dermal papilla cell.

The overexpression of *LOC108637151* was confirmed through transfecting the pcDNA3.1-*LOC108637151* vectors in DPCs (Figure 7A). Further, we found that the overexpression of *LOC108637151* led to a significant upregulation of *SEPP1* expression in DPCs (Figure 7B). These results suggested that *SEPP1* might be a target gene of *LOC108637151* with an establishment of a positive regulatory relationship between *SEPP1* and *LOC108637151*. Additionally, the overexpression of *LOC108637151* resulted in a significant upregulation of the cell proliferation marker genes *Ki67* (Figure 7C) and *PCNA* (Figure 7D), which was further supported by the enhanced proliferation of DPCs from the CCK-8 assay (Figure 7E). These observations suggest that the overexpression of *LOC108637151* facilitate the proliferation of DPCs (Figure 7).

## DISCUSSION

The growth of hair follicles in cashmere goats exhibits periodic rhythmic changes. The development of SHFs in Liaoning Cashmere goats can be categorized into five distinct stages: pre-anagen (April to July), anagen (August to November), early catagen (January), catagen (February) and telogen (February to March) [3]. As the growth cycle of SHFs in cashmere goats, cashmere shedding occurs during the late telogen and early anagen phases. Consequently, Cashmere shedding not only signifies the end of the SHF growth cycle, but also the beginning of a new cycle. The cashmere shedding is closely related to the production of cashmere in cashmere goats, but its molecular regulatory mechanism is not fully understood. The morphological examination of skin tissue sections from cashmere goats can provide crucial morphological insights. Although theoretically the number of SHFs should remain constant throughout the year, researchers have suggested that variations in SHFs numbers are observed due to differences in SHFs depth during each period and slight variations in the location of skin sections [22].

In this study, paraffin-embedded sections of skin tissue from Liaoning Cashmere goats were prepared using Sacpic staining. Analysis of these sections revealed a higher activity and greater number of SHFs in the NS skin tissue slices compared to the AS skin tissue slices. Furthermore, the SHFs in the NS samples were positioned significantly deeper and exhibited a greater width of hair bulbs than those in the AS samples. These findings suggest that the majority of SHFs in NS cashmere goats remained in the telogen phase and had not yet transitioned to the subsequent development cycle. In contrast, most SHFs in AS cashmere goats appeared to have completed the previous hair follicle cycle and were gradually entering the next developmental phase. It is important to note that these observations pertain to the majority of SHFs, not all.

Current studies have demonstrated that *BMP2*, *BMP4* and *Wnt10a* play a significant role in the periodic development of hair follicles, with their expression levels exhibiting distinct characteristics across different periods. Specifically, the expression level of *BMP2* is relatively low during the anagen phase of hair follicles in humans and mice, with minimal significant expression, whereas it significantly increases during the telogen phase of hair follicle development [18]. Additionally, in cashmere goats, *BMP2* plays a crucial role in regulating the cyclical growth of cashmere by inhibiting hair follicle development and maintaining SHFs in the resting phase during hair follicle morphogenesis [23]. The expression patterns of *BMP4* during hair follicle development were found to align with those of *BMP2*, both of which function as inhibitors of hair follicle regeneration, thereby maintaining hair follicles in the telogen phase [19,24]. *Wnt10a* plays a crucial role in the developmental and growth cycles of hair follicles, with its expression being up-regulated during the anagen phase [20]. Recent studies have demonstrated that *Wnt10a* exerts a regulatory influence on the formation and development of hair follicles in fetal cashmere goats, moreover, it is identified as a key gene in controlling the stages of fetal skin development and maturation in cashmere goats, promoting the homeostasis of epithelial cells and dermal fibroblasts through the regulation of *chi-miR-130b-3p* [25,26]. Consequently, these findings provide additional evidence that *BMP2*, *BMP4*, and *WNT10a* could serve as indicator genes distinguishing the telogen and anagen stages in the hair follicle growth cycle of cashmere goats.

In our study, the majority of SHFs in NS cashmere goats remained in the telogen phase and had not yet transitioned to the subsequent development cycle, while in contrast, most SHFs in AS cashmere goats appeared to have completed the previous hair follicle cycle and were gradually entering the next developmental phase. To further validate this observation, we conducted RT-qPCR analysis to assess the relative expression levels of *BMP2*, *BMP4*, and *WNT10a*. We observed that the relative expression levels of *BMP2* and *BMP4* exhibited a progressively decreasing trend from the telogen group to the NS group, AS group, and anagen group. Notably, the relative expression levels of *BMP4* in the telogen and anagen phases were significantly different (p<0.01). Conversely, the relative expression of *WNT10a* exhibited a progressively increasing trend, with a significant difference observed between the telogen and anagen phases (p<0.01). Taken together with the skin histomorphology observations, these findings indicated that the majority of SHFs in NS goats remained in the telogen phase and had not yet initiated the subsequent development cycle, whereas most SHFs in AS cashmere goats had completed the previous hair follicle cycle and were gradually entering the next developmental phase.

Transcriptome sequencing technology (RNA-seq) provides researchers with valuable insights into gene expression levels and transcript structures across temporal and spatial dimensions, thereby facilitating the discovery of previously unrecognized transcripts, rare transcripts, and alternative splicing sites [27]. It significantly enhances our understanding of the transcriptome. In recent years, it has become increasingly evident that ncRNAs also play critical roles in many biological processes. Consequently, transcriptome sequencing now includes the detection of both coding and ncRNAs, providing a more comprehensive view of gene expression. This advancement opens new avenues for investigating the genetic basis of livestock traits at the transcriptional level.

In recent years, numerous researchers have studied the significant influence of ncRNAs and their target genes on the morphogenesis and development of hair follicles. For instance, Ma et al [28] cultured DPCs and fibroblast cells from cashmere goats and conducted high-throughput transcriptome sequencing, revealing that lncRNAs may function as competing endogenous RNAs (ceRNAs) to indirectly regulate hair follicle stem cells during the hair follicle growth cycle. *LncRNA-000133* might be involved in the induction characteristics of DPCs, so it was speculated that it might be related to the formation and growth of cashmere fibers [29]. *LncRNA-599547* was found to promote *Wnt10* expression through sequestering miR-15b-5p, thereby promoting the inductive properties of DPCs of cashmere goats [30]. LncRNA *FABP_AS* acted as chi-miR-335-5p sponge, thereby suppressing hair follicle stem cells proliferation [31]. The melatonin-responsive *lncRNA018392* accelerated the skin fibroblasts cell cycle of cashmere goat and promoted cell proliferation by recruiting *SPI1* to upregulate the expression of the neighboring gene *CSF1R* [32]. LncRNA *MTC* might binds to p65 protein, thereby activating NF-κB signaling pathway to promote the proliferation of skin fibroblasts in cashmere goat [33]. LncRNA *MSTRG.20890.1* could inhibit the proliferation and directional migration of dermal fibroblasts through the chi-miR-24-3p/ADAMTS3 signaling axis, thereby inhibiting the formation of DP structure at embryonic stage [34]. LncRNA *MSTRG.14227.1* can function as a sponge of chi-miR-433, thereby alleviating the inhibitory effect of chi-miR-433 on its target gene *ADAMTS3* [35]. These results suggested that lncRNA plays an important role in the development, cycle and regeneration of SHFs in cashmere goats. In this study, we identified key lncRNAs and genes related to cashmere shedding in goats along with their interacting regulatory relationships. Although the potential biological significance of the identified key lncRNAs and genes needs to be further clarified in SHFs physiological processes of cashmere goats, our results provided a basis for revealing the potential molecular mechanisms of cashmere shedding in goats.

Previously, it was demonstrated that the SEPP1, is expressed in various tissues and abnormal expression of *SEPP1* can result in irregular hair growth in mammals [36–38]. Researchers found the lack of *SEPP1* in epidermal cells might led to the development of hyperplastic epidermis and aberrant hair follicle morphogenesis, accompanied by progressive alopecia after birth [39,40]. In this study, the *SEPP1* was identified as a key candidate gene potentially related to cashmere shedding in cashmere goats. Thus, we strongly recommend that the potential effect of *SEPP1* on cashmere shedding should be further confirmed through appropriate techniques in cashmere goats. On the other hand, here, although skin tissues from both the AS and NS groups were collected in early April, during the telogen phase of SHFs, a greater proportion of hair follicles in the AS group were in the anagen phase compared to those in the NS group. Our results demonstrated that the expression level of *LOC108637151* was higher in the AS group than in the NS group. Moreover, we further confirmed that an increased expression level of *LOC108637151* promotes the proliferation of DPCs, which may facilitate the cashmere shedding in goats. The profound molecular regulatory mechanism remains to be further explored.

## CONCLUSION

Our results indicated that the density, depth, width of SHFs, and the active S/P follicle ratio were significantly lower in the AS cashmere goat skin tissue compared to the NS cashmere goats. The majority of SHFs in NS cashmere goats remained in the telogen phase and had not yet entered the subsequent development cycle, whereas most SHFs in AS cashmere goats had completed the previous hair follicle cycle and were gradually initiating the next development cycle. Key 450 genes and 352 lncRNAs were screened that might be related to cashmere shedding in goats. Furthermore, both the lncRNA *LOC108637151* and its co-expression *SEPP1* exhibited high expression levels in the AS group. The overexpression of *LOC108637151* in DPCs up-regulated the *SEPP1* expression, and promoted the proliferation of cashmere goat DPCs *in vitro*. These findings suggested that they might play a crucial role in regulating the cashmere shedding in goats.

## Figures and Tables

**Table 1:** Growth parameters of hair follicle in both AS and NS cashmere goats

Statistical indexes	AS	NS
SHFs density (N/mm^2^)	7.10±1.98^b^	15.89±2.93^a^
PHFs density (N/mm^2^)	1.03±0.30^a^	0.90±0.33^a^
SHFs depth (μm)	1,274.52±69.43^b^	1,515.14±127.25^a^
SHFs width (μm)	58.17±12.06^b^	76.053±10.84^a^
Active S/P (%)	7.53±3.23^b^	20.07±8.53^a^
Number of active SHFs (N)	6.00±1.68^b^	20.57±2.84^a^
Active SHFs density (N/mm^2^)	2.48±0.70^b^	8.49±1.17^a^

Data on the cashmere performances and parameters related to hair follicle by Duncan test.

a,b In the same row of data, the same shoulder letters represent no significant differences, and different shoulder letters represent significant differences.

AS, already-shed; NS, non-shed; SHFs, secondary hair follicles; PHFs, primary hair follicles.

**Table 2:** The comparison of the obtained sequencing data with the goat reference genome

Sample name	AS_1	AS_2	AS_3	NS_1	NS_2	NS_3
Total reads	82928200	84022218	90776022	80812918	79863544	104494920
Total mapped	80253330 (96.77%)	81077347 (96.5%)	86968940 (95.81%)	77855666 (96.34%)	76915613 (96.31%)	100408477 (96.09%)
Multiple mapped	6471081 (7.8%)	5668195 (6.75%)	7962472 (8.77%)	7183976 (8.89%)	8022812 (10.05%)	9784774 (9.36%)
Uniquely mapped	73782249 (88.97%)	75409152 (89.75%)	79006468 (87.03%)	70671690 (87.45%)	68892801 (86.26%)	90623703 (86.73%)
Read-1	36948351 (44.55%)	37803859 (44.99%)	39590298 (43.61%)	35414965 (43.82%)	34529105 (43.24%)	45414480 (43.46%)
Read-2	36833898 (44.42%)	37605293 (44.76%)	39416170 (43.42%)	35256725 (43.63%)	34363696 (43.03%)	45209223 (43.26%)
Reads map to ‘+’	36845849 (44.43%)	37656759 (44.82%)	39438122 (43.45%)	35292513 (43.67%)	34386516 (43.06%)	45246826 (43.3%)
Reads map to ‘−’	36936400 (44.54%)	37752393 (44.93%)	39568346 (43.59%)	35379177 (43.78%)	34506285 (43.21%)	45376877 (43.42%)
Non-splice reads	58114223 (70.08%)	60272401 (71.73%)	61096434 (67.3%)	55690381 (68.91%)	50974556 (63.83%)	70788943 (67.74%)
Splice reads	15668026 (18.89%)	15136751 (18.02%)	17910034 (19.73%)	14981309 (18.54%)	17918245 (22.44%)	19834760 (18.98%)
Reads mapped in proper pairs	72009162 (86.83%)	73544706 (87.53%)	77110478 (84.95%)	69002474 (85.39%)	67101688 (84.02%)	88498502 (84.69%)

AS, already-shed; NS, non-shed.

## Data Availability

Upon reasonable request, the datasets of this study can be available from the corresponding author. Sequence data that support the findings of this study had been deposited in the GenBank Sequence Read Archive (SRA) database under accession number PRJNA1227581.

## SUPPLEMENTARY MATERIAL

**Supplement 1**. The primer sequences of genes used for quantification in this experiment.

**Supplement 2**. Analysis of lncRNAs’ distribution and features.
