# Supplementary material for: Transcriptome analysis reveals the key long non-coding RNAs and genes related to cashmere shedding in goats
Source: Anim Biosci. 2025 Oct 22;39(3):250499. doi: 10.5713/ab.25.0499 (PMC12963749; doi:10.5713/ab.25.0499)
Supplement: Supplementary file 1 [file ab-25-0499-Supplementary-1.pdf]

Supplement 1. The primer sequences of genes used for quantification in this experiment

Gene name	Primer sequence
GAPDH	F: GCAAGTTCCACGGCACAG<br>R: TCAGCACCAGCATCACCC
β-actin	F: GGCAGGTCATCACCATCGG<br>R: CGTGTTGGCGTAGAGGTCTTT
BMP4	F: GCGAGCCATGCTAGTTTGATACC<br>R: GTGGAAGCTCCTCACGGTGTTG
BMP2	F: CCTTTATATGTGGACTTCAGTG<br>R: GCCTTGGGAATCTTAGAGTTA
Wnt10a	F: CACAGCACCTACCCTCTTGG<br>R: TCACTCTACATCCAGCACCTTG
C7	F: CTGGCTGAGTCATTCTGGAAGGAAC<br>R: ACTGGTCAATCAATCTTCGGTAGGC
DCLK1	F: CTCCTCCACTTCACTTGCATCCAC<br>R: TCCTCCGACACTTCACCTTCTCC
THFAIP6	F: TATGGGAAGAGGCTCACGGATGG<br>R: ATATTCACAGACCGCCTTCGCTTC
DUSP1	F: CCACCACCACTGTCTTCAACTTCC<br>R: GCTGGGAGAGGTCGTGATAGGG
ATF3	F: CGCCATCCAGAACAAGCACCTC<br>R: CTCGGCTTTGGTGACTGACATCTC
FAT4	F: GACGACGGAGTGGCATCATTCAG<br>R: TGTGTTCTGGGAAAGGCAGTTGAC
FHL1	F: TGCCTCAAGTGCTTCGACAAGTTC<br>R: CGTGCCAGTAGCGGTTCTTATAGTG
PLN	F: AAGTCCAGTACCTCACTCGCTCTG<br>R: CTGGAGGTTTTGACGTGCTTGTTG
SFRP4	F: CGCTCACGGATGATGCTTCTCG<br>R: CCTGCTGTTCGCTTCTTGTCCTG
GREM1	F: ACCATCATCAACCGCTTCTGCTAC<br>R: CAGGACAGTTGAGCGTGACCATC
