# Supplementary material for: Transcriptome analysis reveals the key long non-coding RNAs and genes related to cashmere shedding in goats
Source: Anim Biosci. 2025 Oct 22;39(3):250499. doi: 10.5713/ab.25.0499 (PMC12963749; doi:10.5713/ab.25.0499)
Supplement: Supplementary file 2 [file ab-25-0499-Supplementary-2.pdf]

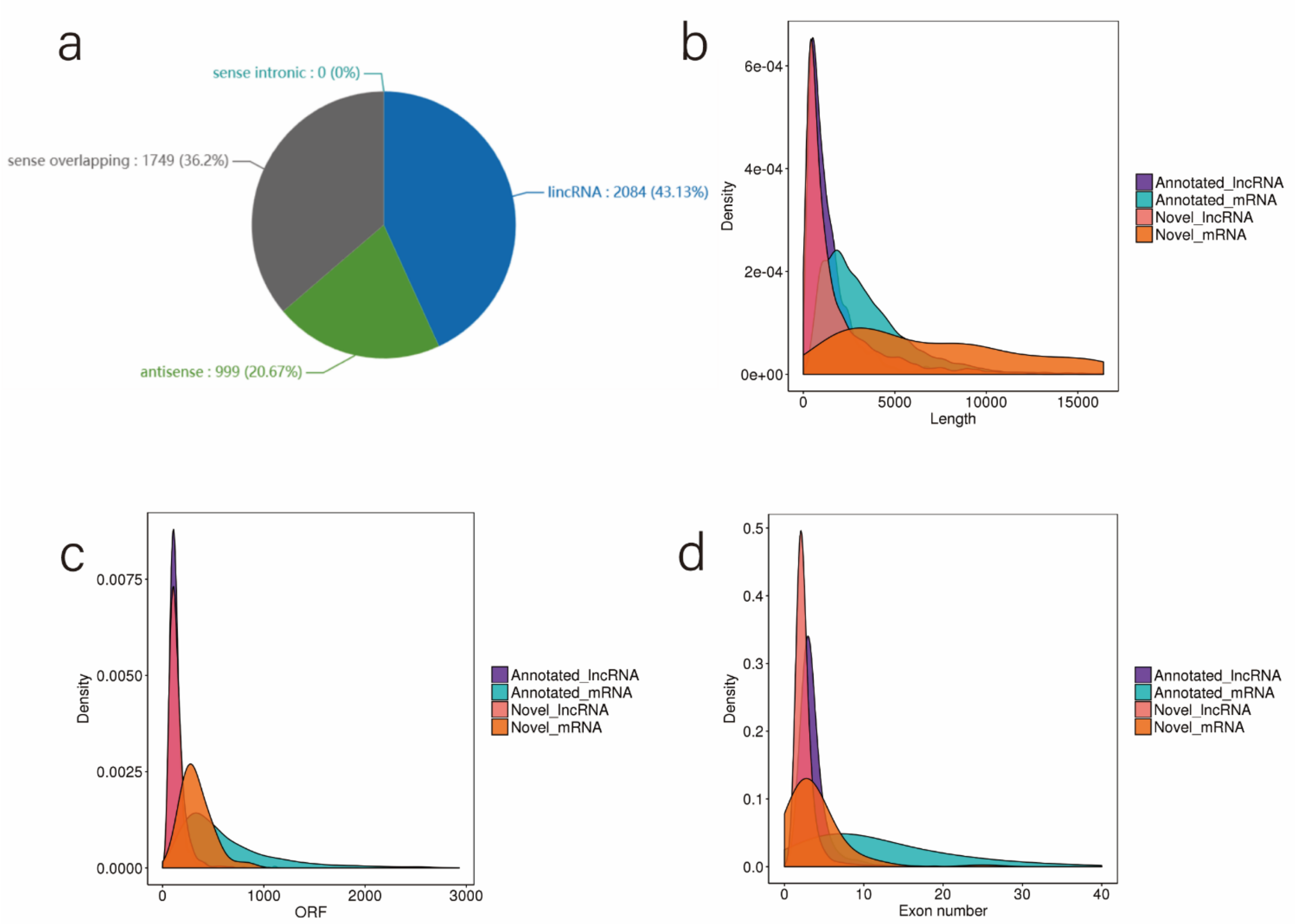

Supplement 2. Analysis of lncRNAs' distribution and features. (a) Distribution map of lncRNAs' type. (b), (c) and (d) Comparative density distribution map of lncRNA and mRNA.
